# *Clostridium difficile* ribotypes in humans and animals in Brazil

**DOI:** 10.1590/0074-02760150294

**Published:** 2015-12

**Authors:** Rodrigo Otávio Silveira Silva, Maja Rupnik, Amanda Nádia Diniz, Eduardo Garcia Vilela, Francisco Carlos Faria Lobato

**Affiliations:** 1 Universidade Federal de Minas Gerais, Escola de Veterinária, Belo Horizonte, MG, Brasil; 2 University of Maribor, Faculty of Medicine, Maribor, Slovenia; 3 National Laboratory for Health, Environment and Food, Maribor, Slovenia; 4 Center of Excellence for Integrated Approaches in Chemistry and Biology of Proteins, Ljubljana, Slovenia; 5 Universidade Federal de Minas Gerais, Faculdade de Medicina, Belo Horizonte, MG, Brasil

**Keywords:** C. difficile, pseudomembranous colitis, zoonosis

## Abstract

*Clostridium difficile* is an emerging enteropathogen responsible for pseudomembranous colitis in humans and diarrhoea in several domestic and wild animal species. Despite its known importance, there are few studies about*C. difficile* polymerase chain reaction (PCR) ribotypes in Brazil and the actual knowledge is restricted to studies on human isolates. The aim of the study was therefore to compare *C. difficile*ribotypes isolated from humans and animals in Brazil. Seventy-six *C. difficile* strains isolated from humans (n = 25), dogs (n = 23), piglets (n = 12), foals (n = 7), calves (n = 7), one cat, and one manned wolf were distributed into 24 different PCR ribotypes. Among toxigenic strains, PCR ribotypes 014/020 and 106 were the most common, accounting for 14 (18.4%) and eight (10.5%) samples, respectively. Fourteen different PCR ribotypes were detected among human isolates, nine of them have also been identified in at least one animal species. PCR ribotype 027 was not detected, whereas 078 were found only in foals. This data suggests a high diversity of PCR ribotypes in humans and animals in Brazil and support the discussion of *C. difficile* as a zoonotic pathogen.

*Clostridium difficile* is an emerging enteropathogen responsible for most cases of pseudomembranous colitis in humans and diarrhoea in several animal species (Songer 2010). In the last years, studies showed a high similarity between *C. difficile* isolates from humans and animals, suggesting a possible zoonotic transmission (Hensgens et al. 2012).

There are few studies about *C. difficile* polymerase chain reaction (PCR) ribotypes in Brazil and the actual knowledge is restricted to studies on human isolates in the state of Rio de Janeiro (Balassiano et al. 2012). Studies showed a high prevalence of *C. difficile*infection (CDI) in piglets and there are reports also in foals, dogs, and wild animals in Brazil (Cruz Junior et al. 2013, Silva et al. 2013a, b, 2014a), but the *C. difficile* PCR ribotypes in domestic animals are still unknown. The aim of the study was therefore to compare *C. difficile* ribotypes isolated from humans and animals in Brazil.

Seventy-six *C. difficile* isolates from humans (n = 25), dogs (n = 23), piglets (n = 12), foals (n = 7), calves (n = 7), one cat, and one manned wolf isolated in between 2008 and 2015 were included (Table I). Human samples were collected from inpatients with suspicious CDI from the University Hospital of the Federal University of Minas Gerais (UFMG) (Silva et al. 2014c). The samples from diarrhoeic dogs, foals, calves, and maned wolf (*Chrysocyon brachyurus*) were obtained directly from the rectum, at the Veterinary Hospital of UFMG at the time of the consultation, and were only collected from animals for which the main motivation for the consultation was the occurrence of diarrhoea (Silva et al. 2013a,b). Samples from apparently healthy dogs and one healthy cat, belonging to students from the university, were collected at the time of defecation (Silva et al. 2013a). The piglets included in this study were submitted to the Veterinary School of UFMG for routine diagnosis of piglet neonatal diarrhoea (Cruz-Junior et al. 2013). A/B toxins were detected by cytotoxicity assay (Silva et al. 2013b) or with a commercial ELISA kit (*C. difficile* Tox A/B II; Techlab Inc, USA). All procedures were previously approved by the Research Ethical Committee of UFMG (CAAE - 0710.0.203.0000.11).

**TABLE I:** Distribution by host and clinical details from 76 *Clostridium difficile* isolates from humans and animals in Brazil

Host	Clinical history	Isolates n (%)	Total n (%)
Humans	CDI	20 (26.3)	25 (32.9)
	Diarrhoeic but negative for A/B toxin	5 (6.6)	
Dogs	CDI	2 (2.6)	23 (30.3)
	Diarrhoeic but negative for A/B toxin	16 (21)	
	Not diarrhoeic	5 (6.6)	
Piglets	CDI	8 (10.5)	12 (15.8)
	Diarrhoeic but negative for A/B toxin	3 (3.9)	
	Not diarrhoeic	1 (1.3)	
Foal	CDI	4 (5.3)	7 (9.2)
	Diarrhoea	3 (3.9)	
Calves	Diarrhoea	8 (10.5)	8 (10.5)
Cat	Not diarrhoeic	1 (1.3)	1 (1.3)
Maned wolf	Diarrhoeic but negative for A/B toxin	1 (1.3)	1 (1.3)

CDI: *C. difficile* infection.

Intergenic spacer regions were amplified using Bidet primers as previously described (Janezic & Rupnik 2010). PCR ribotypes for which the reference strains were available are designated by international Cardiff/Leeds nomenclature, while others are designated by internal nomenclature (SLO and number).

The Table II summarises the PCR ribotypes and clinical history of the *C. difficile* strains included in this study. Twenty-four PCR ribotypes were identified, where 014/020, 009, and 106 were the most common, accounting for 14 (18.4%), 13 (17.1%) and eight (10.5%) samples, respectively. Fourteen different PCR ribotypes were detected among human isolates; nine of them have also been identified in at least one animal species. This data support the discussion of*C. difficile* as a zoonotic pathogen. Also, the present study is the first to report the isolation of *C. difficile* strains positive for binary toxin gene (*cdtB*) in humans in Brazil.

**TABLE II:** Ribotypes and host clinical details from 76 *Clostridium difficile* isolates from humans and animals in Brazil

Ribotype		Total isolates n (%)	Host	Clinical history (number of isolates)
001/072	A^+^B^+^CDT^-^	3 (3.9)	Human	CDI (2)
			Piglet	Not diarrhoeic (1)
009	A^-^B^-^CDT^-^	13 (17.1)	Human	Diarrhoea (1)
			Dog	Diarrhoea (3)
				Not diarrhoeic (2)
			Cat	Not diarrhoeic (1)
			Calf	Diarrhoea (5)
			Foal	Diarrhoea (1)
010	A^-^B^-^CDT^-^	3 (3.9)	Human	Diarrhoea (1)
			Dog	Diarrhoea (2)
011/049	A^+^B^+^CDT^-^	1 (1.3)	Piglet	Diarrhoea (1)
012	A^+^B^+^CDT^-^	3 (3.9)	Piglet	CDI (3)
014/020	A^+^B^+^CDT^-^	14 (18.4)	Human	CDI (4)
			Dog	CDI (2)
				Diarrhoea (3)
				Not diarrhoeic (1)
			Piglet	CDI (1)
				Diarrhoea (2)
			Foal	CDI (1)
050(CE)	A^+^B^+^CDT^-^	1 (1.3)	Human	Diarrhoea (1)
053	A^-^B^-^CDT^-^	4 (5.3)	Dog	Diarrhoea (2)
			Calf	Diarrhoea (1)
			Foal	Diarrhoea (1)
078	A^+^B^+^CDT^+^	3 (3.9)	Foal	CDI (3)
084(CE)		1 (1.3)	Piglet	CDI (1)
106	A^+^B^+^CDT^-^	8 (10.5)	Human	CDI (3)
				Diarrhoea (1)
			Dog	Diarrhoea (3)
				Not diarrhoeic (1)
126	A^+^B^+^CDT^+^	2 (2.6)	Piglet	CDI (2)
131	A^+^B^+^CDT^-^	1 (1.3)	Human	CDI (1)
602(CE)	A^+^B^+^CDT^-^	1 (1.3)	Dog	Diarrhoea (1)
SLO002	A^-^B^-^CDT^-^	6 (7.9)	Human	Diarrhoea (4)
			Dog	Diarrhoea (1)
			Maned wolf	Diarrhoea (1)
SLO046	A^+^B^+^CDT^-^	1 (1.3)	Piglet	CDI (1)
SLO147	A^+^B^+^CDT^-^	2 (2.6)	Human	CDI (1)
			Foal	Diarrhoea (1)
SLO179	A^-^B^-^CDT^-^	1 (1.3)	Calf	Diarrhoea (1)
SLO197	A^+^B^+^CDT^+^	1 (1.3)	Human	CDI (1)
SLO198	A^+^B^+^CDT^+^	2 (2.6)	Human	CDI (1)
			Calf	Diarrhoea (1)
SLO199	A^+^B^+^CDT^-^	2 (2.6)	Human	CDI (1)
			Dog	Diarrhoea (1)
SLO224	A^+^B^+^CDT^+^	1 (1.3)	Human	CDI (1)
SLO225	A^+^B^+^CDT^-^	1 (1.3)	Human	Diarrhoea (1)
SLO231	A^+^B^+^CDT^+^	1 (1.3)	Dog	Not diarrhoeic (1)

CDI: *C. difficile* infection.

PCR ribotype 014/020 was previously reported in three humans with confirmed CDI (Balassiano et al. 2009, Secco et al. 2014) and in free-living coatis (*Nasua nasua*) in an urban park in Brazil (Silva et al. 2014b). At this time, it was again detected in four hospitalised humans with confirmed CDI. Moreover, the PCR ribotype 014/020 was also found in six dogs, three piglets, and in one foal, four of them with CDI, which suggest a high frequency of this PCR ribotype in humans and animals in Brazil. PCR ribotype 014/020 is currently the main cause of CDI in the European community and has been reported in animals in several countries including Germany, Netherlands, United States of America, and Slovenia (Bauer et al. 2011, Janezic et al. 2012, 2014). Thus, the present study corroborates previous reports that showed PCR ribotype 014/020 can colonise a broader range of species and also it is present in different geographic regions, although this type is not recognised as being hypervirulent (Janezic et al. 2014).

Among toxigenic strains, PCR ribotype 106 was the second most frequent in this study. Until 2009, PCR ribotype 106 was reported only in the United Kingdom when then it was identified in one human with CDI (Balassiano et al. 2009) in Brazil and recently detected also in coatis (Silva et al. 2014b). In the present study, 106 has been identified in humans (4 isolates) and dogs (4 isolates), suggesting that this PCR ribotype might be common in humans and also in animals in the country.

The PCR ribotype 009 (also known as *53-like*) was the second most common in the present study. This type was found in 13 (17.1%) isolates, one from human and four from different animal species, which corroborates previous studies (Janezic et al. 2012) and suggest that this PCR ribotype, similar to 014/020, is common and might have a high capacity to colonise different species. Among eight isolates from calves, five were classified as PCR ribotype 009. Considering that the strains were isolated from calves between 10-60 days of age from five different farms, the present result suggest a low diversity in this group compared with the other species or with previous studies with strains from calves (Janezic et al. 2012, 2014, Koene et al. 2012,Knight et al. 2013). Finally, it is also interesting to note that recent studies have shown that nontoxigenic strains has a potential to prevent CDI in humans and piglets (Songer et al. 2007, Oliveira Junior et al. 2016, Zhang et al. 2015). Considering that the nontoxigenic ribotype 009 has a high capacity to colonise different species, this may be a good candidate for future studies focusing on CDI prevention by colonisation with nontoxigenic strains.

Some PCR ribotypes previously reported in humans in Brazil (Balassiano et al. 2009, 2010,2011) were not identified in the present study, including PCR ribotypes 038, 133, 135, and 233. Together with the present report, these results suggest a high diversity of PCR ribotypes in humans and animals in Brazil, similar to previously reported in other countries. Moreover, the prevalence of some PCR ribotypes appears to vary in different geographical regions. Also, the present study is the first to report the isolation of *cdtB* in humans in Brazil.

It is remarkable that some common PCR ribotypes in Europe and North America, such as 002, 015, 045, and 150, seem to be rare in Brazil. Recently, some reports suggest the rapid emergence of the epidemic 027 strain in Latin America, with confirmed cases in Costa Rica, Panama, and Chile (Hernández-Rocha et al. 2012, Quesada-Gómez et al. 2012, López-Ureña et al. 2014). Anyway, this *C. difficile* ribotype is still not reported in animals and humans in Brazil. PCR ribotypes 078 has also drawn the attention of researchers for its increased frequency in cases in humans and/or animals (Hensgens et al. 2012). Again in contrast with these findings, 078 were found only in three foals (4%) with confirmed CDI. The present study suggests that Brazil has some marked differences in the pattern of *C. difficile* ribotypes in humans and animals compared to those reported in the rest of the world. Anyway, considering the continental dimensions of the country and the limited number of isolates evaluated, these results should be analysed cautiously.

This is the first study to evaluate *C. difficile* strains from domestic animals in Brazil and shows a high diversity of PCR ribotypes, also with a considerable number of ribotypes present in both humans and various domestic animals.
